# Differential Effects of Resistance- and Endurance-Based Exercise Programs on Muscular Fitness, Body Composition, and Cardiovascular Variables in Young Adult Women: Contextualizing the Efficacy of Self-Selected Exercise Modalities

**DOI:** 10.3390/medicina57070654

**Published:** 2021-06-25

**Authors:** Sime Versic, Kemal Idrizovic, Gentiana Beqa Ahmeti, Damir Sekulic, Matej Majeric

**Affiliations:** 1 Faculty of Kinesiology, University of Split, 21000 Split, Croatia; simeversic@gmail.com; 2 Faculty for Sport and Physical Education, University of Montenegro, 81400 Niksic, Montenegro; kemo@t-com.me; 3 Faculty of Physical Education and Sport, University of Prishtina, 10000 Prishtina, Kosovo; gentiana.beqa@uni-pr.edu; 4 Faculty of Sport, University of Ljubljana, 10000 Ljubljana, Slovenia; matej.majeric@fsp.uni-lj.si

**Keywords:** physical exercise, strength, flexibility, blood pressure, women

## Abstract

There is an evident lack of research simultaneously investigating endurance training (ET) and resistance training (RT) with regard to their potential influence on fitness and health status in young women. This study aimed to determine the effects of RT and ET three times a week over an eight-week period on anthropometric/body composition indices, blood pressure (BP), and muscular fitness in apparently healthy young women who participated in a self-preferred program. The sample of participants comprised 57 young healthy women (23.9 ± 3.08 years, 165.5 ± 5.8 cm, 66.8 ± 7.2 kg) divided into ET (*n* = 18), RT (*n* = 19), and non-exercising (C) (*n* = 20) groups. The variables consisted of anthropometric/body composition indices (body mass, BMI, body fat, and lean body mass), muscular fitness variables (lower body strength, upper body strength, abdominal strength, dynamometric force, and flexibility), and cardiovascular parameters (resting heart rate and systolic and diastolic BP). A pre- and post-testing design, with factorial analysis of variance for repeated measurements (ANOVA: Group × Measurement), including a consecutive post hoc test, was applied. The ANOVA indicated a similar improvement in body composition (increase in lean body mass and decrease in body fat percentage), resting heart rate, and flexibility in both of the exercise groups, with no significant changes in the C group. RT improved the participants’ strength and force capacities to a greater extent than ET. BP showed a trend of improvement in both of the training groups, but without statistically significant pre- to post-changes. Correlation analysis calculated with variables of pre- to post-differences (VDs) indicated poor associations between VDs, indicating relative independence of the obtained effects for the different variables in each training group. Although this investigation comprised apparently healthy young women, numerous positive changes indicated the efficacy of both programs in a relatively short period of time. While the participants in this study self-selected training programs, the evidenced positive effects can be at least partially related to this fact.

## 1. Introduction

Cardiovascular diseases (e.g., coronary artery disease, stroke, and heart failure) are the leading causes of death worldwide, with increased blood pressure (BP)/hypertension (higher than 120 mmHg and 80 mmHg for systolic (SBP) and diastolic (DBP), respectively) as one of the most important indicators of an increased risk [1,2,3]. Pharmacological drugs are used as a standard means of lowering BP and have no significant side effects [4]. However, mainly for economic reasons, a healthy lifestyle that includes diet control, reduced cigarette and alcohol consumption, and, above all, physical exercise, is recommended as the most optimal way of disease prevention [4,5,6]. Therefore, there is an evident increase in interest about the potential efficacy of various types of physical exercise for reducing BP levels in different population groups [5,7]. 

Another important indicator of health status is physical fitness. Specifically, physical fitness is considered an integrated measure of most of the body functions involved in the performance of daily physical activity [8], while muscular fitness, including flexibility and strength, is recognized as the most important facet of physical fitness in adulthood [9]. Namely, better strength and flexibility are known to be associated with a lower risk of the development of diseases, injury, and functional disability [10,11]. Optimal strength levels are important for performing everyday activities, because all movements of the locomotor system are based on muscle function [11]. Meanwhile, apart from being related to overall musculoskeletal functioning, flexibility (i.e., range of motion in joints) is associated with the occurrence of pain and injury [12]. Not surprisingly, the world-leading authority in sports medicine, American College of Sports Medicine (ACSM), highlights the importance of strength and flexibility in everyday life and for proper functioning [12]. 

For the purpose of the prevention of cardiovascular diseases and the maintenance/improvement of muscular fitness, physical exercise is of the utmost importance. Physical exercise is a form of physical activity performed with a specific goal and is accordingly planned and structured [13]. These goals are mainly focused on inducing changes in anthropometric/body composition indices (i.e., decrease in fat mass and increase in lean body mass) and improvements in muscular fitness (i.e., strength, flexibility, and balance), which together have a positive impact on cardiovascular health and overall health status [14,15]. Physical exercise comes in many different structures and forms, with resistance training, endurance training, and a combination of the two types as the most commonly practiced [13,16]. In brief, endurance training involves a cyclical type of activity (e.g., running, swimming, and cycling) and primarily affects the respiratory and cardiovascular systems [17,18]. On the contrary, resistance training is a form of exercise with an external load (i.e., own body weight, free weights, machine-derived resistance, and elastic bands), and this form of training mostly develops an individual’s muscular fitness status, although effects on cardiovascular indices are also reported [5,19,20]. 

Although the effects of resistance and endurance training vary, especially when intensity, extent, external load shape, and other training variables are considered, research has shown that both training modalities effectively reduce SBP and DBP [4]. For example, in a meta-analysis of 47 clinical trials that investigated the effects of aerobic exercise on resting SBP and DBP in adults, relative decreases of 4% (SBP) and 5% (DBP) in hypertensive and 2% (SBP) and 1% (DBP) in normotensive adults have been reported [21]. Similar findings were reported in another meta-analysis that included 28 randomized, controlled trials with resistance training, where a decrease in BP was registered in most of the observed normotensive and hypertensive groups [7]. In addition to the confirmed effects on the BP parameters, both endurance and resistance training affect body composition and fitness status [14,22]. Specifically, studies on women of different ages have found that resistance training is an effective strategy to reduce body fat, which is another important cardiovascular risk factor, while both endurance and resistance training have been shown to be an effective way to reduce subcutaneous adipose tissue and body mass index (BMI) [16,23,24].

Irrespective of the fact that positive effects of both endurance- and resistance-based training are frequently reported, there is an evident lack of research simultaneously investigating differential effects of these two types of training with regard to their potential influence on fitness and health status in young women [15]. From our perspective, this is particularly important because of the known fact that being a male has significantly higher positive association with involvement in physical exercise [25]. For example, if we compare employed men and women, authors of the study are of the opinion that women at the same time face greater parental responsibilities and home duties than men [26]. This altogether limits their possibility to be engaged in some form of PE. On the contrary, maintaining a regular optimal level of physical exercise is extremely important for women’s overall motor functioning, but also for the prevention of the development of diseases characteristic of later life stages, such as osteoporosis [27,28].

Therefore, this study aimed to determine the effects of eight-week exercise programs on anthropometric/body composition indices, BP, and muscular fitness in apparently healthy young women. These information will give clear practical implications for planning and optimizing the training regimes in this population in order to achieve morphological, health and fitness goals. We hypothesized that both programs would induce positive changes on the studied variables, with: (i) superior effects of resistance training on muscular fitness capacities (strength, flexibility, and dynamometric force), and (ii) superior effects of endurance training on BP and anthropometric/body composition status.

## 2. Methods

### 2.1. Participants and Design of the Study

The sample of participants in this study consisted of 57 young healthy women (age: 24 ± 3 years; initial body height: 165 ± 6 cm; body mass: 66.8 ± 7.2 kg; BMI: 24.37 ± 2.57 kg/m^2^) who were members of one fitness center in Prishtina, Kosovo. The participants were mostly university students and did not have any previous experience regarding physical exercise and training. They were divided in two experimental groups—resistance training group (RT; *n* = 19), and endurance training group (ET; *n* = 18), and a non-exercising control group (C; *n* = 19). Initially, groups consisted of more participants (ET = 21; RT = 22, and C = 20), but only those who participated in >80% of training sessions (for training groups) both testing sessions (all groups), and who were not involved in some physical exercising during the study period (C group) were observed in the study, resulting in drop-out rate of 11%.

**Figure 1:**
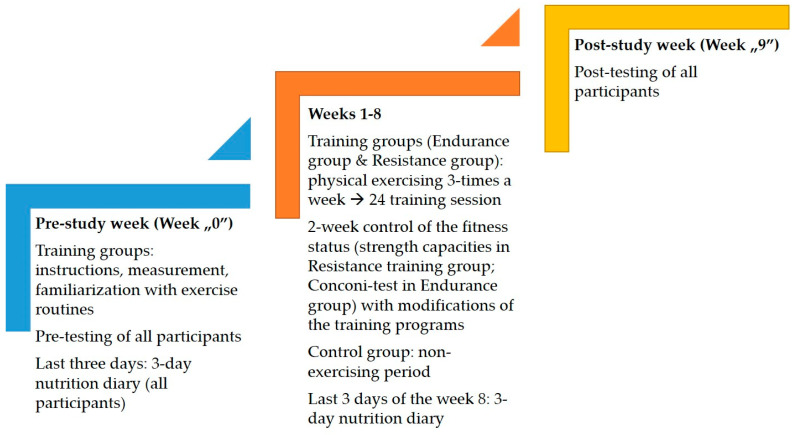
Study design and characteristics of the study phases.

As a certain methodological novelty of the study, we must highlight the fact that the participants were not randomly allocated to one of the groups, but division was carried out on the basis of participants’ self-preferences. Namely, all participants self-decided to initiate physical exercising in the same fitness center, where the authors of this study offered them different forms of PE. The participants then chose one of the proposed forms of PE. Even women in the control group expressed interest in exercising, but due to a lack of space in the groups, they were unable to participate immediately and, therefore, were observed as the “non-exercise group” over the eight-week period. The study design is presented in Figure 1.

We must note that the participants were included in the study at different dates (when they personally decided to start the physical exercise program), but all of them were observed in the period between mid-January and early May 2020. The study purpose and aim and the benefits and risks were explained to all of the participants who confirmed participation in the study by signing informed consent forms. This study was approved by the Ethical Board of the University of Split, Faculty of Kinesiology, Split, Croatia (EBO: 2141-6775-234).

### 2.2. Variables

Testing was organized twice: (i) pre-testing before the intervention and (ii) post-testing after the eight-week training regime (Figure 1). The set of variables consisted of anthropometric/body composition indices, muscular fitness variables, and cardiovascular parameters.

The anthropometric/body composition variables included body height (BH), body mass (BM), body mass index (BMI), body fat percentage (BF%), and lean body mass (LBM). BH was measured by a Seca stadiometer (Seca, Birmingham, UK). BM, BF% and LBM were measured with a bioimpedance device (Tanita TBF-300, Tanita, Tokyo, Japan). BMI was calculated as the ratio of BM (kg), and squared BH (m). These measurements were done in the morning and the participants were instructed not to eat before. The reliability of the measurement indicated throughout intra-class coefficient of the correlation (ICC) for anthropometric/body composition variables on the basis of test-retest protocol ranged from 0.96 (for BH), to 0.82 (for LBM and BF% measurement). Muscular fitness was evaluated by an assessment of flexibility and strength capacities. The participants were familiarized with all of tests over several familiarization training/testing sessions that were carried out in the “null week” of the experiment (Figure 1).

Flexibility was evaluated by the sit-and-reach test (S&R) and the shoulder circumduction test (SCT). In the S&R test, the participants were in a seated position without shoes, legs fully extended and feet resting on the front of a wooden box on which the upper side was a marked scale and sliding indicator. On signal from the examiner, the participants had to bend over their upper body with stretched legs and push the indicator as far as possible, holding the final positions for 2 s. The test was repeated three times and the best result was noted as the final result. For the SCT test, the participants held the measuring stick with both hands in front of their face and had to perform maximal, both-arm shoulder circumduction with their hands and arms fully extended. The distance between the palms was measured and the goal was to make circumduction with the smallest distance possible. Three trials were carried out, and the best result (the smallest distance between palms) was recorded as the final result for each participant. The ICC value of 0.93 evidenced high reliability of the flexibility measurement.

Strength capacities included dynamometric force of the right and left hands, squat, sit-up, and push-up tests. Dynamometric forces were measured with a calibrated hand-grip dynamometer (Lafayette Manual Muscle Tester, Model # 01163, Lafayette Instrument Inc., Lafayette, IN, USA). The participants had to hold the dynamometer in their hand, with their arm extended, and move it away from the body, squeezing it maximally for at least three seconds. The test was repeated two times for both sides and the better results, expressed in kilograms, were noted down (ICC = 0.82, and 0.80 for right and left hand, respectively). The squat test was performed with a time limit, as the participants needed to make the maximal number of squats in 30 s. The participants started the test in the standing position in front of a chair, looking in the opposite direction, and had to squat down and touch their backside on the chair and then return to the standing position with legs fully extended. The number of repetitions performed in 30 s was recorded. In the push-ups, the participants performed modified push-ups with their knees and hands as base support. The task was to lower the chest to the ground and then raise it again on outstretched arms, repeating until complete exhaustion. The final result was expressed as the maximal number of repetitions. The maximal number of sit-ups was used as a test of abdominal dynamic strength. The participants laid on a mat with their knees bent, feet flat on the floor and hands crossed on the chest. The tester held the participants’ feet on the ground, and the participants performed their maximal number of sit-ups by touching their knees with the elbows.

The cardiovascular variables included SBP, DBP, and resting heart rate. For the BP and heart rate monitoring, a manual sphygmomanometer (Omron M3, Omron, Kyoto, Japan) was used. The measurements were conducted by an experienced technician in the morning prior to anthropometry testing. The participants were in a seated position, with parallel legs and their back and arm supported at the heart level. They were instructed to be in a relaxed position and to avoid talking.

At the beginning and at the end of the study, the participants were asked to write their nutrition intake in food diaries, 3 days at the beginning and at the end of the study, and the data was later analyzed with nutritional tables and software.

### 2.3. Training Programs

The training interventions lasted eight weeks and included a total of 24 training sessions (three times per week). This duration was selected based on the authors’ expertise in women’s fitness habits, as a two-month period represents the usual time before significant drop-outs occur. The inclusion criteria for the participants was a minimum of 21 training session conducted. All training sessions were carried out in a fitness center in Prishtina, Kosovo, organized with a one-day rest between them, and lasted between 45 and 60 min.

The ET group trained on Nova 450 treadmills (Nova Sport, Istanbul, Turkey) with dimensions of 56 × 150 cm, which allowed the participants to adjust their walking/running speed (from 1 to 20 km/h) and the inclination of the surface (from 0% to 15%). During the “null week” of the experiment (Figure 1), the participants in the ET group conducted a Conconi test in order to estimate their anaerobic threshold (pre-testing value of 154 ± 11 beats per minute). This test is typically used as a non-invasive, indirect method to determine endurance capacities, with a monitoring association between running speed and heart rate [29]. The heart rate values at anaerobic thresholds allowed individualization of training, as live monitoring was carried out with heart rate chest belts. The participants had to perform all training elements with their heart rate in the range of 5–30 beats below the anaerobic threshold, and to achieve the given tasks, they could change their running speed and the incline. Continuous, interval, and fartlek running protocols were explained to the participants, who were instructed to perform one type of training each week, but also with the option to change the protocol every training session. Given the adaptations of the cardiorespiratory system, a Conconi test was conducted every two weeks in order to adjust the threshold and optimal training loads.

The RT group performed circuit weight training with exercises using their own body weight, handheld weights, and weight machines (Technogym, Cesena, Italy). Before the start of the intervention, all of the participants conducted two introductory training sessions in order to learn the proper techniques and to become familiarized with the equipment, and in the same time training sessions, the instructors noted the optimal weights for each exercise (“null week”; please see Figure 1). The participants performed three sets with progressive load increase and continued until failure in the final set. These weights were revised every two weeks as the participants conducted additional individual testing training sessions, led by one of the authors. Training were carried out in groups of four to six participants, and each had an individualized training load for each exercise. Exercise was conducted with details about repetitions, sets, and order, as presented in Table 1.

### 2.4. Statistics

Since the Kolmogorov-Smirnov test indicated all of the variables as normally distributed, descriptive statistical analysis in the study included means and standard deviations. Levene’s test served for checking the homoscedasticity of the variables.

For determining the differences between the groups and the measurements in the observed variables, a two-way ANOVA for repeated measurements was applied for the group (C, ET, and RT) and measurement (pre- and post-measurement). Moreover, Scheffe’s post hoc analysis was conducted. To present the effect size (ES), the partial eta squared values (η^2^) were calculated (small ES: >0.02; medium ES: >0.13; large ES: >0.26). To present the changes in each variable for each group, differences between pre- and post-measurement are presented in percentages.

In order to evaluate the associations between the obtained changes, we calculated the variables of the differences (VD) for each observed anthropometric/body composition, muscular fitness, and cardiovascular parameter (outcomes) by subtracting the pre- and post-test results for each outcome and each participant. Consequently, higher numerical value presented greater change in studied outcome. Pearson’s correlation was used to evaluate the associations between the VDs, which allowed us to identify the associations between changes (i.e., qualitative effects) that occurred as a result of the applied interventions.

For all calculations, Statistica 13.5 (TIBCO Software Inc., Palo Alto, CA, USA) was used, with a significance level of *p* < 0.05.

## 3. Results

Main ANOVA effects for “Group” were evidenced for body fat (small ES), lean body mass (small ES), squats 30 s (medium ES), and push-ups (medium ES). Main effects for “Measurement” were statistically significant for all variables (with large ES), but SBP and DBP. Finally, significant factor of “Interaction” was found for all variables but SBP and BDP, with medium ES for sit and reach, and large ES for remaining variables (Table 2).

As evidenced from data on descriptive statistics and post hoc ANOVA analysis, control group improved performance in sit and reach test of flexibility significantly (*p* < 0.05). Both training groups improved: body composition significantly (increased lean body mass and decreased body fat percentage), decreased body mass and BMI, increased flexibility (both for sit and reach and shoulder circumduction), and achieved better results in push-ups. Resistance-training group additionally improved the performance in sit-ups, 30 s squats, and dynamometric force tests (Table 3).

**Figure 2:**
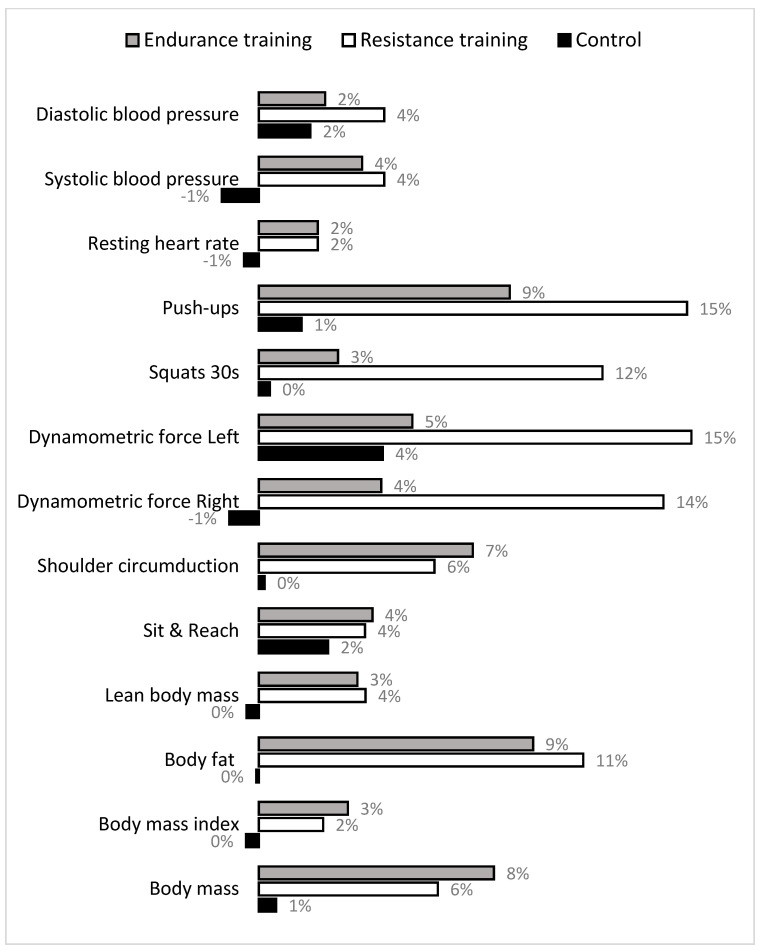
Percentage differences (improvements) in observed variables between pre- and post-measurement.

Changes between pre- and post-measurement are informatively presented in Figure 2, where pre- to post-differences in study variables are presented in percentages. Evidently, the most evident improvements (evidenced as “positive %”) in both training groups occurred in strength capacities, and dynamometric force (with superior training effects in RT group), and body composition variables (with similar improvements in both training groups). Positive trends of changes in both training groups is evidenced for variables of cardiovascular status also.

Correlations between VD are presented in Supplementary Tables S1–S3. Evidently, VDs better correlated in C (Supplementary Table S1), then in RT (Supplementary Table S2) and ET (Supplementary Table S3) (thirteen, five and seven significant coefficients of correlation, respectively), indicating relative independence of changes in tested variables for training groups. However, it is generally interesting to note that changes in SBP and DBP were correlated in both training groups (Pearson’s correlation: 0.49, and 0.50 for RT and ET, respectively). Additionally, changes in body mass significantly correlated with changes in BP variables, and push-up performance in ET (Pearson’s correlation: 0.62, 0.46, and 0.65, respectively).

Data obtained by nutritional diaries (please see Methods for details), indicated no significant differences in caloric intakes at baseline (before the study), and follow-up (end of the study period) measurement, for any of the study groups (RT: 2354 ± 311 vs. 2401 ± 441 kcal; ET: 2561 ± 451 vs. 2511 ± 439 kcal; C: 2601 ± 561 vs. 2722 ± 602 kcal, for baseline and follow-up, respectively).

## 4. Discussion

This study aimed to investigate the differential effects of eight-week interventions of resistance- and endurance-training in healthy adult women. Accordingly, there are several important findings. First, positive changes in body composition and most of the observed muscular fitness variables were evidenced in both experimental groups. Second, the RT group achieved better improvements in muscular fitness compared to the ET group. Finally, while the resting heart rate significantly decreased in both exercise groups, the BP level showed a decreasing trend without statistically significant changes.

### 4.1. Anthropometry/Body Composition

The participants in both experimental groups noted progress in the variables of body composition as a result of the training programs. In both groups, changes were evidenced as (i) a decrease in BF% and (ii) an increase in LBM. We may say that the decrease in BF% was expected and should mostly be explained by an increased metabolism of stored fat cells and an increased caloric expenditure that occurred as a result of exercise [30]. On the contrary, the increase in LBM probably occurred as a result of adaptation to the external load to which participants were exposed, which first included neural adaptations accompanied by a gradual increase in myofibrillar proteins, consequently leading to muscular hypertrophy [24]. However, given the different forms of exercise, the results of endurance and resistance training should be observed and discussed separately.

In general, endurance training is regularly recommended as an effective approach for reducing BF% [14]. Therefore, our results are in accordance with previous studies that investigated the effects of this type of exercise in women [31,32]. For example, in a 12-month randomized, controlled clinical trial, sedentary participants performed an average of 295 min/week home-based moderate- to vigorous-intensity aerobic activity [31]. The results showed a significant decrease in body mass, BMI, and body fat, while changes were more evident in individuals with a higher average weekly training volume [31]. Similar results were observed in obese premenopausal women who exercised four to five times a week for 90 min at approximately 55% of their maximal aerobic power over a period of 14 months [32]. After the intervention, an average body fat mass drop of 4.6 kg was recorded [32]. Although such changes are more noticeable in obese women due to a higher ceiling for improvement, studies have confirmed the positive effects of endurance training, even in young healthy women [33,34]. For example, in a study consisting of a two-month endurance exercise program performed by indoor cycling, a decrease in body fat in young healthy females was also noted [33].

The participants in the endurance group experienced an increase in LBM. Knowing the characteristics of endurance training (i.e., relatively low intensity), such findings may seem surprising. A possible explanation may be found in the fact that the sample consisted of women of low training status, so even the relatively low external load, but repetitively performed due to running, caused certain muscle adaptations, mostly in the lower extremities. Although an increase in LBM is not often seen in endurance training, some studies in which interventions have been performed using indoor cycling have achieved similar results [22,35]. For example, in a study on middle-aged and older women, intensive cycling training showed similar advances in lean body mass gain compared to resistance and combined resistance endurance training [22]. Therefore, although there is no doubt that the external load was higher in the RT, it seems that the large number of repetitions in ET resulted in hypertrophy of the involved musculature in our participants, probably because of their low training status.

Resistance training caused positive changes in anthropometric/body composition variables, evidenced as decrease in BF%, as well as an increase in LBM. As presented in the Methods Section, the circuit resistance training used in this study involved a number of exercises with an external load that involved either the participants’ own body weight (i.e., squats, push-ups, and curl-ups) or some equipment-based exercises (i.e., dumbbells, machines). Circuit training is usually designed in such a way that each exercise is repeated over a certain time period (or for certain number of repetitions), and after a rest, the participant moves on to the next exercise. Although primarily oriented toward an improvement in muscular strength and endurance, due to training characteristics, involving the work of a large number of muscle groups with short breaks, resulting in high heart this form of physical exercise was efficient in inducing changes in anthropometric/body composition indices due to circuit-type training and a relatively high energy expenditure [23,36]. In a study on healthy but sedentary young women in Brazil, a significant drop in BF% was noted due to a 10-week intervention composed of circuit strength training [37]. Similar results have been reported in studies in obese women, so we can say that the results of our study are in line with the expectations [38].

### 4.2. BP and Heart Rate

A meta-analysis on the effects of BP and BP-regulating mechanisms found that a drop in exercise pressure occurs through a reduction in vascular resistance, in which the sympathetic nervous system and the renin-angiotensin system play a key role [39]. The sympathetic nervous system is one of the three basic parts of the autonomic nervous system and is responsible for, among other things, controlling the cardiovascular system during physical exercise [40]. The renin-angiotensin system is a hormonal system that regulates BP, fluid and electrolyte balance, and systemic vascular resistance [41]. Studies examining the effects of physical exercise on various organ systems have noted a significant decrease in plasma renin and sympathetic nervous system activity [42]. Therefore, it is generally accepted that both endurance and resistance training have a positive effect on lowering BP without significant differences between the two types of training [4].

However, our results did not show a significant decrease in BP measures as a result of the applied training programs, which is not supportive of our initial hypothesis. There are two potential reasons for such results. First, the interventions lasted eight weeks only, which is a shorter duration than in studies that previously reported a significant reduction in BP in exercising participants [4]. Specifically, although the results of similar studies suggest that, due to reduced control over a longer period of time, the duration of an intervention has a negative effect on lowering BP, studies confirming the positive effect of various types of physical exercise on BP usually last between 12 and 24 weeks [4]. The second explanation could be found in the fact that we observed healthy young women who were (mostly) categorized as normotensive at the study baseline, and thus had a low ceiling for improvement. Other studies support this, having confirmed that BP reductions after endurance and resistance training are greater in hypertension patients compared to those with normal BP and hypotension [4].

Meanwhile, the observed decrease in resting heart rate (HR) emerged as an important positive adaptation to the applied training programs. Since previous studies have regularly noted an increase in stroke volume and a decrease in resting HR after training, our findings are in line with the expectations [39]. For example, a study on healthy sedentary men and women noted a decrease in resting HR, which was more pronounced with a higher intensity [43]. It is interesting that both endurance and resistance training induced similar positive changes in resting HR, which is intriguing knowing that endurance-based training is generally considered as being more effective for improvements in the cardiovascular status of participants [14].

Indeed, resistance training is definitively not the first choice of exercise when the aim is a positive impact on resting HR, but the characteristics of the applied resistance training program (circuit weight training) probably contributed to the positive effects of this type of physical exercise on resting HR. Specifically, in circuit weight training, a large number of muscles involved and a short rest between exercises cause an increase in HR and consequently lead to adaptation of the cardiovascular system [36]. Supportively, the authors of the study, which compared the effects of traditional strength training and circuit strength training, recorded a higher average HR during circuit training and concluded that it may be an effective training strategy for the promotion of both strength and cardiovascular adaptations [44]. In addition, studies have shown that long-term resistance training increases parasympathetic activity and decreases sympathetic activity directed toward the human heart during rest [45]. As a consequence, athletes have a lower resting heart rate and a more rapid heart rate recovery due to enhanced parasympathetic activity [46].

### 4.3. Muscular Fitness

Given the involvement of different organ systems in RT and ET, differential effects of these two types of training were expected. Specifically, ET primarily depends on the ability of the respiratory and cardiovascular systems to deliver oxygen to the muscles to be used to perform specific work [47,48]. On the contrary, RT depends on the synchronized action of the muscular and nervous systems to create internal tension in response to external load [49]. As a direct consequence, superior changes in strength are expected as a result of RT [14,20,50].

The evidenced improvement in strength capacities in the RT group is in line with our initial expectations, as their training was based on strength exercises. Previous studies that have followed the effects of resistance training in women are consistent with such findings, as strength progress almost always occurs as a result of systematic and planned work with external loads [23,51,52]. A study investigating physiological adaptations to strength and circuit training in postmenopausal women noted advances in isometric strength and upper and lower limb dynamic strength after 24 weeks of circuit strength training [52]. Moreover, in a study on obese women, progress due to the implementation of circuit strength training was recorded in the measures of maximum and repetitive strength [52]. However, studies have rarely examined the changes in various strength capacities of young, apparently healthy females. Our results are, therefore, encouraging, especially considering that the experimental training program we applied here was relatively short (i.e., eight weeks).

Although it may seem surprising, the ET group improved their results in the push-up test. However, it is almost certain that the improved scores in the push-up strength test emerged as a consequence of anthropometric/body composition changes. Namely, the endurance group recorded a decrease in BM and BF% after the eight-week training intervention. With these changes, the participants actually made it easier for themselves to perform the test as they had to lift a smaller load. This is directly supported by results of a correlation analysis, where changes (pre-post-differences) in the push-up test were significantly correlated with the changes evidenced for body mass, with a better improvement in sit-ups among those participants who reduced their mass to a greater extent. In support of this, research on a large sample of young men showed that BM and BF% correlated negatively with muscular endurance measured with push-ups, sit-ups, and repeated squats [53].

Progress in flexibility occurred due to the organizational specifics of training in both experimental groups, i.e., stretching exercises in the warm-up and cool-down sections of the training. Namely, during the warm-up part of the training, the participants carried out preparation exercises for the main part of the training session (which included raising body temperature), as well as activated and increased their range of motion through mobility and dynamic flexibility exercises. Moreover, at the end of each training session, regardless of whether it was endurance or resistance training, the participants conducted a cool down that included static stretching exercises. Numerous studies have unequivocally confirmed that stretching exercises, regardless of static, dynamic, or some other type of stretching, have a positive effect on range of motion and flexibility [54,55]. Although dynamic flexibility exercises are regularly considered as being more effective in the development of flexibility [56,57], our results actually support the idea that static stretching can be considered as an effective method for flexibility development in female participants of low-training status [58,59].

Although the control group did not achieve as significant improvement in flexibility as the training groups, even the significant improvement in sit and reach test in the control group is surprising, given that the group did not perform any intervention. The background of such a result can be explained in two ways. In the first instance, it is possible that the sit and reach test is not reliable or independent of unsystematic errors. This may primarily be due to variations in the measured characteristics or the fact that some participants were able to artificially improve their score by performing flexibility exercises immediately before testing. Indeed, research has shown that stretching exercises have an acute effect in terms of range of motion [60,61]. Another potential explanation is the hypothetical framework. Namely, as explained in the Methods Section, the participants in the control group were initially interested to join some type of PE, but due to lack of space or equipment, they did not have the opportunity to participate in the desired training program. Since they were motivated for PE, it is possible that during the study period, they themselves engaged in some activity that included stretching exercises, which resulted in an improvement in flexibility. However, since they were not instructed about performing some type of training, and they did not do it so systematically, they could not achieve improvements in the other measured variables.

### 4.4. Limitations and Strengths

There are several limitations of this study. Most importantly, the daily activity and nutrition of the participants were not constantly monitored, and there is a possibility that these habits affected some of the observed variables. However, it seems that the dietary habits of the participants did not change during the course of the study. Moreover, we did not use randomization for the experimental and control groups, and the participants were allocated to training groups on the basis of their own preferences regarding the type of PE. Yet, this could be viewed as a strength of the study, because it actually mimics a real-life situation in which a person will definitively choose a training program on the basis of her own preference. The menstrual cycle was not controlled before and after the training program. Since the cyclic variations of estrogen and progesterone levels during a menstrual cycle affect some variables of physical fitness this could be viewed as study limitation. One of the limitations of the study is use of Tanita TBF-300 for body composition assessments, which has questionable validity and measurement error and may lack the precision to assess small changes in body composition [62]. However, we believe that with the same measurment protocol and pre-test guidelines, measurement errors were minimized. Finally, this study lacks comparison of pre-testing between group differences in aerobic capacity. Therefore, there is a certain possibility that initial differences between groups at least partially influenced even the training effects.

A major strength of this study is the fact that it is one of the first studies exploring the differential effects of endurance and resistance training on the anthropometric, motor, and cardiovascular parameters in young healthy women. Additionally, the relatively controlled training programs, and regular evaluation of fitness status are important strengths of this investigation. Given the context and characteristics of the participants, we think that these results can be generalized across the population of young healthy women.

## 5. Conclusions

Although both training groups achieved some improvements in muscular fitness status, our results suggest that resistance training should be observed as a better solution than endurance training with regard to benefits in strength capacity. At the same time, both training groups achieved similar improvements in flexibility measures. As a result, our first hypothesis can be partially accepted.

However, our second study hypothesis should be rejected, as we evidenced no significant differences between the observed training interventions regarding their effects on anthropometric/body composition indices. Namely, although we expected superior effects of endurance-based training on anthropometric/body composition, it seems that resistance training organized as circuit weight training may be an equally effective training method for such a purpose. The fact that the participants in both training groups achieved positive changes in anthropometric/body composition indices is very encouraging, given that we observed young and apparently healthy women. However, it must be stressed that participants self-selected the training programs, and, therefore, it is possible that their personal motivation and satisfaction contributed to the overall training efficacy.

Both types of training similarly affected cardiovascular status, observed throughout resting HR. On the contrary, irrespective of some positive trends, the BP measures did not change significantly in any of the training groups. Therefore, we can conclude that a training program of longer duration is needed in order to achieve positive changes in the BP indices in normotensive women. However, it is evident that even such relatively short training duration of training induced certain positive effects, and therefore may be observed as protective in control of important cardiovascular parameters.

## Figures and Tables

**Table 1:** Plan and program of the resistance-training.

Body Region	Exercise	1st Week	2nd Week	3rd Week	4th Week	5th Week	6th Week	7th Week	8th Week
Abdominals	Abdominal curls	#	#	#				#	
Abdominals	Abdominal curls (added weights)				##	##	##		##
Abdominals	Leg raises							#	
Lower back	Hyperextension bench	#	#	#	##	##	##	#	#
Thigs	Knee extension	#				#			
Hamstrings	Hamstring curl	#	#	#	#	#	#	#	
Legs/gluteus	Lunges		#			#	#		
Legs/gluteus	Lunges (added weights)							#	#
Legs/gluteus/lower back	Squats			#	#		#		
Legs/gluteus/lower back	Squats (plus weights)							#	#
Upper legs	Legg adductions	#	#	#		#			#
Upper legs	Legg abductions	#	#	#			#		#
Upper backs/arms	Latt pulldowns	#	#				#		#
Upper backs/arms	Rowing torso			#	#	#		#	
Arms	Biceps curls	#	#	#	#			#	
Arms	Triceps extension	#	#	#					
Chest	Butterfly machine	#	#					#	
Chest/arms	Bench press			#	#	#	#		#
Chest/arms/shoulders	Inclined bench press								
Shoulders/arms	French press				#			#	#
Number of circuits per training session		2	2	3	3	2	3	3	2
Time of work per set (in seconds)		30	30	25	30	30	30	30	30
Rest between sets (in seconds)		30	30	35	30	30	30	30	30
Rest between circuits (in minutes)		5	5	5	5	5	5	5	5
Warm-up (in minutes)		10	10	10	10	10	10	10	10
Cool-down (in minutes)		10	10	10	10	10	10	10	10

LEGEND: M—weight machines, FW—free weights, BW—own body weight, “#” indicates number of sets for each exercise in one circuit.

**Table 2:** Results of the factorial analysis of variance for repeated measurements, with “Group” (endurance-training, resistance training, and control), and Measurement (Pre- and Post-measurement) as main factors, and Group × Measurement interaction factor (*p*—level of significance, η^2^—effect size).

	Main Effects						Interaction		
	Group			Measurement			Group × Measurement		
	F Test	*p*	ŋ^2^	F Test	*p*	ŋ^2^	F Test	*p*	ŋ^2^
Body mass	0.29	0.76	0.01	374.01	0.001	0.87	77.15	0.001	0.73
Body mass index	0.65	0.52	0.02	147.62	0.001	0.72	63.19	0.001	0.69
Body fat	3.59	0.03	0.11	203.01	0.001	0.78	53.08	0.001	0.65
Lean body mass	4.39	0.01	0.13	26.87	0.001	0.32	9.55	0.001	0.25
Sit and Reach	0.83	0.43	0.02	160.13	0.001	0.73	3.23	0.04	0.11
Shoulder circumduction	4.31	0.02	0.13	71.41	0.001	0.56	18.3	0.001	0.39
Dynamometric force Right	3.05	0.06	0.09	83.3	0.001	0.59	60.76	0.001	0.68
Dynamometric force Left	1.4	0.25	0.05	118.87	0.001	0.67	20.5	0.001	0.41
Squats 30 s	7.13	0.001	0.2	35.45	0.001	0.39	17.42	0.001	0.38
Push-ups	20.85	0.001	0.42	231.58	0.001	0.8	49.59	0.001	0.63
Resting heart rate	0.25	0.77	0.001	27.87	0.001	0.32	14.22	0.001	0.33
Systolic BP	0.61	0.54	0.01	2.69	0.10	0.05	1.7	0.19	0.05
Diastolic BP	0.07	0.93	0.001	2.91	0.09	0.08	0.91	0.41	0.03

**Table 3:** Descriptive statistics (data are given as means ± standard deviations), and post hoc differences of the analysis of variance.

	Control		Resistance Training		Endurance Training	
	Pre-Test	Post-Test	Pre-Test	Post-Test	Pre-Test	Post-Test
Body mass (kg)	65.38 ± 8.32	64.79 ± 9.12	66.56 ± 6.34	60.5 ± 6.42 *	67.35 ± 8.39	59.38 ± 8.56 *
Body mass index (kg/m^2^)	23.77 ± 2.71	24.2 ± 2.49	24.16 ± 2.41	21.98 ± 2.43 *	25.11 ± 2.69	22.09 ± 2.79 *
Body fat (%)	33.94 ± 6.06	34.02 ± 6.22	35.17 ± 5.75	24.19 ± 4.5 *^,C^	36.43 ± 5.43	27.14 ± 4.04 *^,C^
Lean body mass (kg)	42.96 ± 3.87	42.55 ± 3.94	43.29 ± 2.78	46.91 ± 3.99 *^,C^	44.23 ± 4.06	47.57 ± 4.4 *^,C^
Sit and Reach (cm)	25.75 ± 3.26	28.1 ± 3.39 *	26.6 ± 3.9	30.2 ± 4.26 *	25.7 ± 3.87	29.55 ± 3.66 *
Shoulder circumduction (cm)	89.75 ± 8.39	89.75 ± 7.4	82.1 ± 15.21	76.15 ± 15.38 *	87.5 ± 10.47	80.25 ± 10.46 *
Dynamometric force Right (kg)	66 ± 16.44	64.1 ± 16.58	65.55 ± 7.21	79.25 ± 6.12 *^,C,ET^	62.25 ± 9.27	66.4 ± 9.77
Dynamometric force Left (kg)	61.9 ± 13.69	66.1 ± 13.91	60.6 ± 9.33	75.25 ± 5.62 *	60.15 ± 8.99	65.35 ± 9.52
Squats 30 s (reps)	19.46 ± 6.44	19.83 ± 5.73	19.07 ± 6.39	30.71 ± 10.93 *^,C,ET^	16.79 ± 3.76	19.48 ± 4.89
Push-ups (reps)	12.75 ± 3.74	14.2 ± 3.19	15.65 ± 5.59	30.15 ± 8.01 *^,C,ET^	13.55 ± 4.48	22.05 ± 3.78 *^,C^
Resting heart rate (bpm)	72.6 ± 4.82	73.1 ± 4.23	72.95 ± 4.26	70.95 ± 4.26 *	73.3 ± 3.47	71.3 ± 3.47 *
Systolic BP (mmHg)	116.75 ± 8.16	118 ± 6.96	119.5 ± 9.45	115.25 ± 7.86	121 ± 8.05	117.5 ± 7.52
Diastolic BP (mmHg)	81.25 ± 5.82	79.5 ± 6.05	83 ± 6.37	78.75 ± 3.93	81.5 ± 6.9	79.25 ± 5.45

Legend: * indicates significant (*p* < 0.05) within group differences, ^ET^—significantly (*p* < 0.05) different when compared to endurance training group, ^C^—significantly (*p* < 0.05) different when compared to control group.

## Data Availability

The data presented in this study are available on request from the corresponding author.

## Supplementary Materials

The following are available online at https://www.mdpi.com/article/10.3390/medicina57070654/s1, Table S1: Pearson’s correlations between variables of pre- to post-measurement differences for control group, Table S2: Pearson’s correlations between variables of pre- to post-measurement differences for resistance training group, Table S3: Pearson’s correlations between variables of pre- to post-measurement differences for endurance training group.
