# Supplementary material for: Differential Effects of Resistance- and Endurance-Based Exercise Programs on Muscular Fitness, Body Composition, and Cardiovascular Variables in Young Adult Women: Contextualizing the Efficacy of Self-Selected Exercise Modalities
Source: Medicina (Kaunas). 2021 Jun 25;57(7):654. doi: 10.3390/medicina57070654 (PMC8304697; doi:10.3390/medicina57070654)
Supplement: Supplementary file 1 [file medicina-57-00654-s001.zip › medicina-1245575-supplementary.pdf]

Supplementary Table S1. Pearson's correlations between variables of pre- to post-measurement differences for control group (* indicates statistical significance of $p < 0.05$)

	1	2	3	4	5	6	7	8	9	10	11	12
Body mass (1)												
Body mass index (2)	0.66*											
BF% (3)	0.35	0.07										
LBM (4)	0.60	0.35	0.76*									
Sit & Reach (5)	0.33	0.20	0.64*	0.64*								
Shoulder circumduction (6)	0.27	0.23	0.12	0.11	0.04							
Dynamometric force Right (7)	0.67*	0.37	0.80*	0.90*	0.60*	0.13						
Dynamometric force Left (8)	0.67*	0.37	0.80*	0.90*	0.60*	0.13	0.81*					
Squats 30s (9)	0.01	0.17	0.07	0.00	0.09	0.28	0.07	0.07				
Push-ups (10)	0.02	0.00	0.00	0.17	0.05	0.38	0.07	0.07	0.19			
Resting heart rate (11)	0.02	0.06	0.02	0.30	0.32	0.32	0.28	0.28	0.19	0.04		
Diastolic blood pressure (12)	0.14	0.12	0.19	0.18	0.22	0.12	0.19	0.19	0.44	0.16	0.24	
Systolic blood pressure (13)	0.20	0.16	0.09	0.21	0.48*	0.36	0.09	0.09	0.25	0.27	0.27	0.14

Supplementary Table S2. Pearson's correlations between variables of pre- to post-measurement differences for resistance training group (* indicates statistical significance of $p < 0.05$)

	1	2	3	4	5	6	7	8	9	10	11	12
Body mass (1)												
Body mass index (2)	0.87*											
BF% (3)	0.42	0.43										
LBM (4)	0.17	0.23	0.17									
Sit & Reach (5)	0.13	0.27	0.11	0.06								
Shoulder circumduction (6)	0.01	0.15	0.08	0.12	0.02							
Dynamometric force Right (7)	0.30	0.38	0.14	0.07	0.29	0.38						
Dynamometric force Left (8)	0.20	0.28	0.54*	0.11	0.37	0.16	0.48*					
Squats 30s (9)	0.26	0.27	0.09	0.24	0.51*	0.13	0.23	0.39				
Push-ups (10)	0.42	0.35	0.30	0.08	0.22	0.05	0.28	0.28	0.46			
Resting heart rate (11)	0.11	0.10	0.21	0.15	0.09	0.11	0.08	0.21	0.37	0.15		
Diastolic blood pressure (12)	0.26	0.19	0.20	0.09	0.07	0.02	0.03	0.17	0.08	0.03	0.20	
Systolic blood pressure (13)	0.09	0.27	0.09	0.11	0.24	0.10	0.17	0.18	0.20	0.28	0.21	0.49*

Supplementary Table S3. Pearson's correlations between variables of pre- to post-measurement differences for endurance training group (* indicates statistical significance of $p < 0.05$)

	1	2	3	4	5	6	7	8	9	10	11	12
Body mass (1)												
Body mass index (2)	0.75*											
BF% (3)	0.13	0.08										
LBM (4)	0.33	0.27	0.17									
Sit & Reach (5)	0.44	0.37	0.16	0.34								
Shoulder circumduction (6)	0.11	0.24	0.14	0.02	0.39							
Dynamometric force Right (7)	0.33	0.16	0.01	0.29	0.34	0.27						
Dynamometric force Left (8)	0.16	0.11	0.05	0.52*	0.56*	0.22	0.52*					
Squats 30s (9)	0.17	0.08	0.28	0.08	0.48*	0.35	0.02	0.12				
Push-ups (10)	0.45*	0.65*	0.21	0.16	0.42	0.05	0.11	0.02	0.21			
Resting heart rate (11)	0.12	0.02	0.00	0.18	0.11	0.13	0.24	0.21	0.20	0.19		
Diastolic blood pressure (12)	0.35	0.29	0.20	0.05	0.12	0.01	0.39	0.01	0.20	0.07		
Systolic blood pressure (13)	0.62*	0.46*	0.03	0.24	0.27	0.03	0.29	0.10	0.40	0.07	0.16	0.50*
